# Volatile compounds emission from teratogenic human pluripotent stem cells observed during their differentiation *in vivo*

**DOI:** 10.1038/s41598-018-29212-0

**Published:** 2018-07-23

**Authors:** Rosamaria Capuano, Paola Spitalieri, Rosa Valentina Talarico, Alexandro Catini, Ana Carolina Domakoski, Eugenio Martinelli, Maria Giovanna Scioli, Augusto Orlandi, Rosella Cicconi, Roberto Paolesse, Giuseppe Novelli, Corrado Di Natale, Federica Sangiuolo

**Affiliations:** 1 Department of Electronic Engineering, University of Rome Tor Vergata, Via del Politecnico 1, 00133 Rome, Italy; 2 Department of Biomedicine and Prevention, University of Rome Tor Vergata, Via Montpellier 1, 00133 Rome, Italy; 3 Department of Chemical Science and Technology, University of Rome Tor Vergata, Via della Ricerca Scientifica, 00133 Rome, Italy; 4 Centro Servizi Interdipartimentale STA, University of Rome Tor Vergata, Via Montpellier 1, 00133 Rome, Italy

## Abstract

Several investigations point out that the volatile fraction of metabolites, often called volatilome, might signal the difference processes occurring in living beings, both *in* vitro and *in vivo*. These studies have been recently applied to stem cells biology, and preliminary results show that the composition of the volatilome of stem cells *in vitro* changes along the differentiation processes leading from pluripotency to full differentiation. The identification of pluripotent stem cells is of great importance to improve safety in regenerative medicine avoiding the formation of teratomas. In this paper, we applied gas chromatography and gas sensor array to the study of the volatilome released by mice transplanted with human induced pluripotent stem cells (hiPSCs) or embryoid bodies (EBs) derived from hiPSCs at 5 days and spontaneously differentiated cells at 27 day. Gas chromatography analysis finds that, in mice transplanted with hiPSCs, the abundance of 13 volatile compounds increases four weeks after the implant and immediately before the formation of malignant teratomas (grade 3) become observable. The same behaviour is also followed by the signals of the gas sensors. Besides this event, the gas-chromatograms and the sensors signals do not show any appreciable variation related neither among the groups of transplanted mice nor respect to a placebo population. This is the first *in vivo* observation of the change of volatile metabolites released by human induced pluripotent stem cells and hiPSCs-derived cells during the differentiation process. These results shed further light on the differentiation mechanisms of stem cells and suggest possible applications for diagnostic purposes for an early detection of tumor relapse after surgery.

## Introduction

Human induced pluripotent stem cells (hiPSCs), as well as human embryonic stem cells (hESCs), are the only cells able to maintain their cellular identity through self-renewal and, in the meantime, to differentiate into all tissue types. This capability is a hallmark of stemness and a cornerstone for future regenerative medicine. The breakthrough of hiPSCs has raised the possibility that patient-specific iPSCs can provide autologous cells for cell therapy without the concern for immune rejection. However, these traits also make these cells tumorigenic, and consequently hinder the fulfilment of their clinical potential.

The tumorigenic character of these cells plays a central role in defining the optimal hiPSC lines. In fact, teratoma formation *in vivo* is considered the most stringent assay of pluripotency, providing a more reliable and comprehensive confirmation than does testing cells on petri dish *in vitro*. On the other hand, a rigorous safety testing of hiPSCs or hiPSCs- derived cells is imperative to avoid any contamination rescue due to undifferentiated cells leading to teratoma formation^1^.

Teratomas are tumors characterized by a rapid growth *in vivo*, and they are composed by a mixture of tissues remnants of all three germ layers. Teratoma is histopathologically classified as mature (benign tumor) or immature (grades 1–3), and specifically immature teratoma grades 1 and 2 as borderline, and grade 3 as malignant^2^.

In order to develop safe hESC- and hiPSC-based treatments, the tumorigenicity hurdle must be overcome^1^. Three general strategies to cope with this risk have been suggested:

1. terminal differentiation or complete elimination of residual pluripotent stem cells from culture;
2. interfering with tumour-progression genes to prevent tumour formation from the residual pluripotent cells;
3. tumor detection and elimination after its initial formation in the patient’s body.

A further approach for the identification of pluripotent stem cells may be offered by the measure of their metabolic products. Among them, the volatile metabolites (also called volatilome) are particularly attractive because of the expected simplicity of their collection and the large availability of analytical methods suitable for their measurement^3^.

To this regard, we have recently shown that, *in vitro*, each step of the differentiation process of stem cells is characterized by the release of a specific pattern of volatile metabolites. These changes are measurable by both gas-chromatography and gas sensor arrays^4^, and both these instrumental techniques report a large difference between hiPS cells and their successive differentiated phases.

These preliminary results prompted us to investigate if volatile compounds could also detect hiPSCs *in vivo*. To this aim, groups of CD-1 nude mice have been transplanted with cells at different stages of development and specifically with undifferentiated hiPSCs, embryoid bodies derived from hiPSCs at 5 days (EBs) and spontaneously differentiated cells at 27 days (Diff). The profile of volatile compounds released by the animals was sampled weekly, and analyzed using the same experimental approach used for the previous *in vitro* study. The headspace of animal cages was sampled with a solid-phase microextraction probe (SPME) and analysed with a gas chromatograph - mass spectrometer (GC/MS). In parallel, the air of the cage was analyzed by an array of 12 porphyrins functionalized gas sensors^5^. In addition, the gas sensors also analysed the bedding materials of the cages collected at the end of each measurement.

Both GC/MS and sensor arrays are clearly able to detect the appreciable differences in the pattern of volatile compounds occurring at 28 days after cells injection and only in mice implanted with hiPSCs. At that time, 13 volatile compounds shown an abrupt change of concentration. The same event is also recorded by 9 of the 12 sensors. The increase of emission occurs immediately before that teratomas could be visually observed.

These results provide the evidence that the pattern of volatile metabolites changes specifically when undifferentiated cells (hiPSCs) start evolving into an immature teratoma (grade 3). This finding is the first *in vivo* evidence of the prediction of the insurgence of a teratoma after hiPSCs engraftment. For these reasons it may offer a further methodology to improve the safety of stem-cells based therapies.

## Results

### hiPS cells induced immature teratomas when injected subcutaneously into nude mice

The animals injected with hiPS cells have developed a visible tumor mass which has become evident after 28 days. The size of the tumors has been measured immediately before the VOCs measurement from the first day after implant to the 70^th^ day. The body weight was also measured before each measurement session. All animals experienced a moderate increase in the body weight along the experiment. (see Figure S1 in Supplementary Information file).

**Figure 1:**
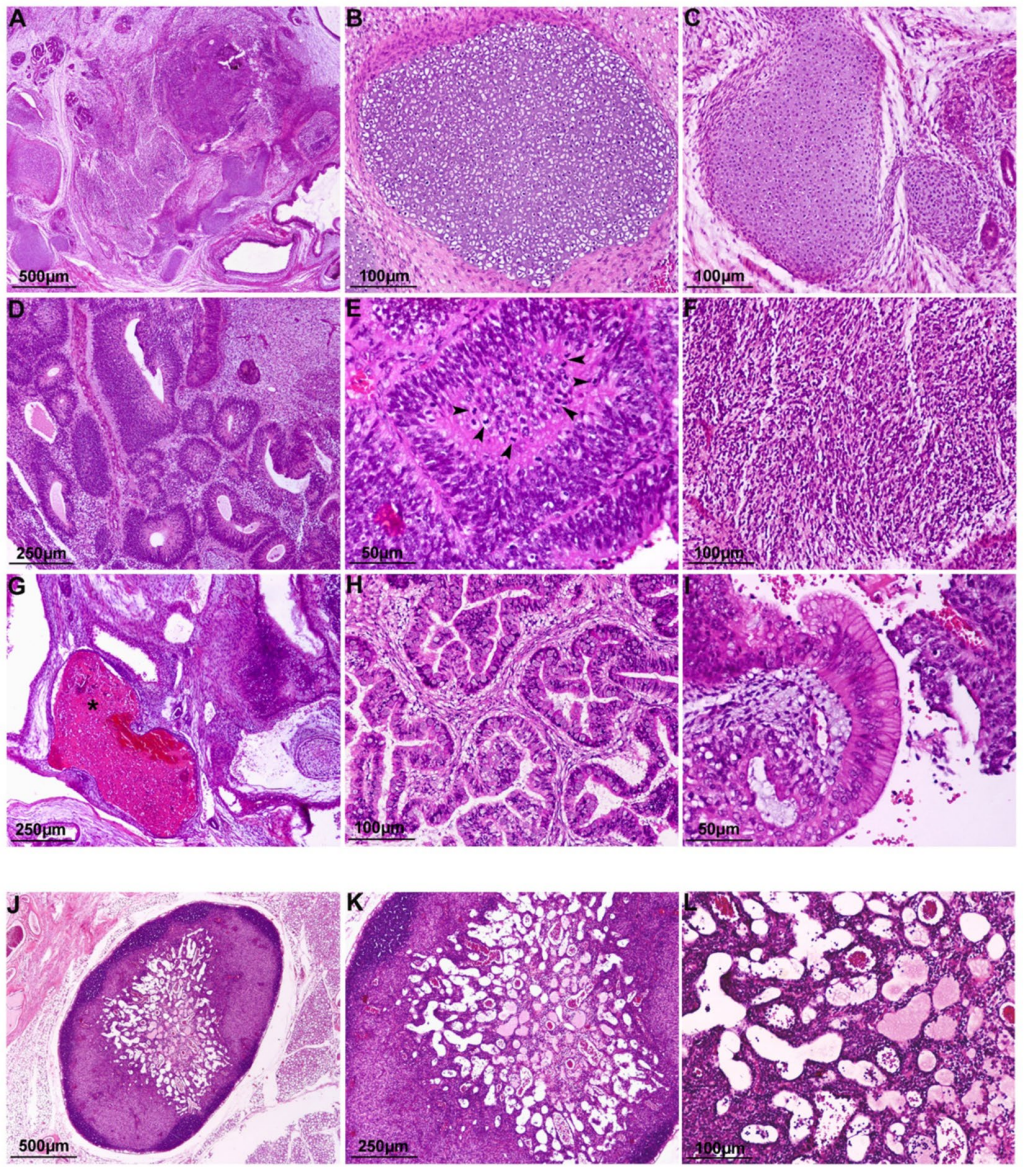
Histological examination of tumors from hiPSCs and EB cells. Representative microscopic images of Haematoxylin & Eosin-stained sections of (**A**) hiPSCs derived-teratoma showing the differentiation into all three germ layers; (**B**,**C**) mature and immature cartilage (mesoderm); (**D,E**) primitive neuroectodermal tissue (endoderm) in the form of rosettes with high mitotic activity (arrowheads); (**F**) cellular primitive stromal tissue; (**G**) hemorrhage and necrosis (asterisk); (**H,I**) glandular and columnar epithelial tissue (endoderm); (**J,L**) a small lymph node associated with fibrosis and edema was found in the site of inoculation of differentiated EBs (20 days).

At the 70^th^ day all animals were sacrificed. Histological examination revealed that hiPS cells induced teratomas and differentiated into all three germ layers: neural tissues (ectoderm), cartilage (mesoderm) and mucosal/ glandular structures (endoderm) (Fig. 1A–I). The presence of immature cartilage (Fig. 1C), primitive neuroectodermal tissue in the form of rosettes with high mitotic activity occupying four or more low-magnification fields in the tumor (Fig. 1D,E), cellular primitive stromal tissue (Fig. 1F), and hemorrhage/necrosis (Fig. 1G), indicated the higher grade of immaturity (malignancy), immature teratoma grade 3. However, no metastases were detected.

The inoculation of EBs (5 days) as well as spontaneously differentiated cells after 27 days (Diff) did not induce any teratoma formation (Fig. 1J–L). Small lymph nodes associates with fibrosis and edema were found in the sites of inoculation in both experimental conditions. No metastases were observed.

### Gas Chromatography/Mass spectrometry

The air samples collected from the cages were analyzed by the combination of SPME (sample collector) and GC/MS (sample analyzer). Empty cages and clean cage bedding materials were also measured and the released compounds were eliminated from the analysis. Eventually, 55 VOCs attributed to the animal metabolism were found (see Table S1 in the Supplementary information file). About half of these VOCs have been episodically observed in less than 70% of all samples. The percentage of occurrence of each VOC is also shown in Table S1.

To ensure a meaningful comparison among samples, the analysis has been restricted to those compounds that was found in more than 70% of all samples. VOCs have been scrutinized to avoid that compounds found exclusively in one group of animals were ruled out. The 22 recurrent VOCs are shown in Table 1. They are almost exclusively linear and branched alkanes and aldehydes containing from 7 to 12 carbon atoms.

**Table 1:** Experimental pipeline.

Cells line	Method of generation	Method of cell detachment and dissociation	Number of mice	Site and number of cells per injection	Teratoma	Time of teratoma formation	Method of analysis
hiPSCs-P1 hiPSCs-V2	hSTEMCCA lentiviral vector	Collagenase IV/accutase solution	5 CD1 Nude	subcutaneous 10 × 10^6^	Immature teratoma Grade 3	4–10 weeks	GC/MS gas sensors H&E
EBs at day 5	floating	Trypsin-like enzyme (TrypLE)	5 CD1 Nude	subcutaneous 5 × 10^6^	lymph nodes, fibrosis and edema in the site of inoculation		GC/MS gas sensors H&E
differentiated cells	spontaneous	Trypsin	5 CD1 Nude	subcutaneous 5 × 10^6^	Lymph nodes, fibrosis and edema in the site of inoculation		GC/MS gas sensors H&E
Placebo	Matrigel		5 CD1 Nude	subcutaneous 100 µl			GC/MS gas sensors

All the recurrent compounds are commercially available, so their identity has been validated by a comparison of the elution time and the mass spectra found in samples with those of standard compounds^6^. The rest of the compounds in Table S1 have only been putatively identified through a comparison with the library databases.

Most of the compounds in Table 1 are typical components of the volatilome of cultured cells and living organisms whose concentration is found altered by several pathologies. Table 1 lists some of the most relevant cases where these VOCs have been found.

**Figure 2:**
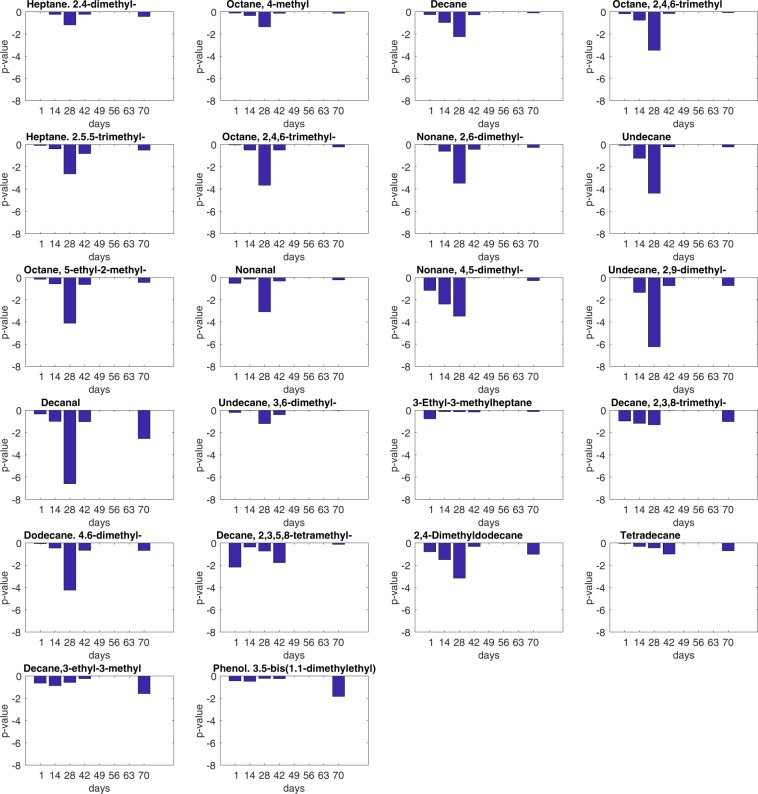
Statistical significance of the abundance of each VOC between mice inoculated with hiPSCs and the other groups calculated at each experimental day.

The abundance of these VOCs changes along the experiment (see Figure S2 in the supplementary information file), however, the behavior of the abundances is rather erratic and does not show any appreciable difference between the four groups with the noteworthy exception of those in which hiPSCs have been injected and only at 28 day from the inoculation. The statistical difference between the abundance released by mice injected with hiPSCs respect to the others has been evaluated with a non-parametric Kruskal-Wallis rank sum test. The results are expressed as the null hypothesis probability (p-value). A comparison between the p-values obtained at each day and for each VOC is shown in Fig. 2.

A statistically significant difference (p < 0.01) is shown at day 28 by 13 of the 22 VOCs. These VOCs and the p-value are marked in Table 1.

The increase of VOCs at day 28 is observed together with the appearance of immature teratomas. Furthermore, the largest increase of the abundance is found in those mice that eventually developed the largest teratomas.

A clear evidence of the behavior of the VOCs abundance is obtained from the principal component analysis (PCA). PCA decomposes a set of multivariate data into non-correlated variables^7^. In practice, PCA introduces a number of novel variables which are linear combinations of the pattern elements. The variance explained by each principal component defines a hierarchy among the principal components, with the assumption that the first principal components carrying the largest variance may faithfully represent the multivariate data and, in particular, the relationship between them.

**Figure 3:**
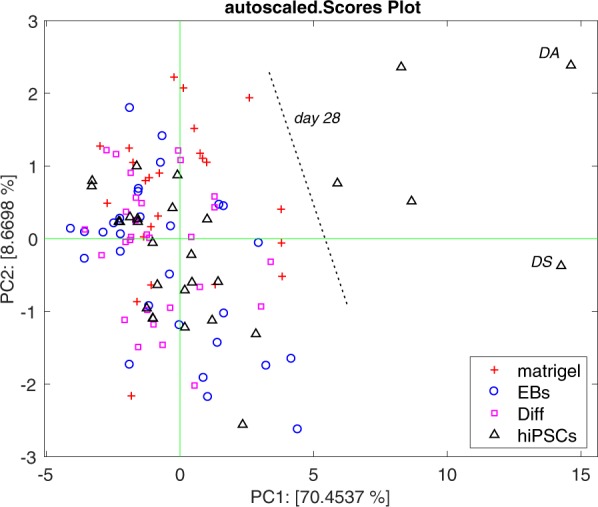
Plot of the first two principal components of the abundance of the 13 compounds showing the increase of the abundance at day 28. The plot accounts for more than 78% of the total variance of the data. No separation among the groups is observed except the hiPSCs at day 28. The largest departure from the average of the distribution is found in those mice, labelled as DA and DS, that eventually developed the largest teratomas.

PCA was applied to the matrix of the abundances of the 13 compounds which are altered at day 28. Figure 3 shows the plot of the first two principal components. The four groups of mice are rather undistinguishable except those injected by hiPSCs at day 28. Since all VOCs increase their abundance at day 28, the segregation of these data occurs along the first principal component. Noteworthy, the largest departure from the average of the data is found for those mice where teratomas of largest size are developed.

### Gas sensor array

A gas sensor array made of 12 porphyrins coated quartz microbalances was applied to measure both the total air sampled from the animal cage and the bedding material of the cages. The air of the cage was measured immediately after the SPME sampling, after that the bedding materials was collected and separately measured.

**Figure 4:**
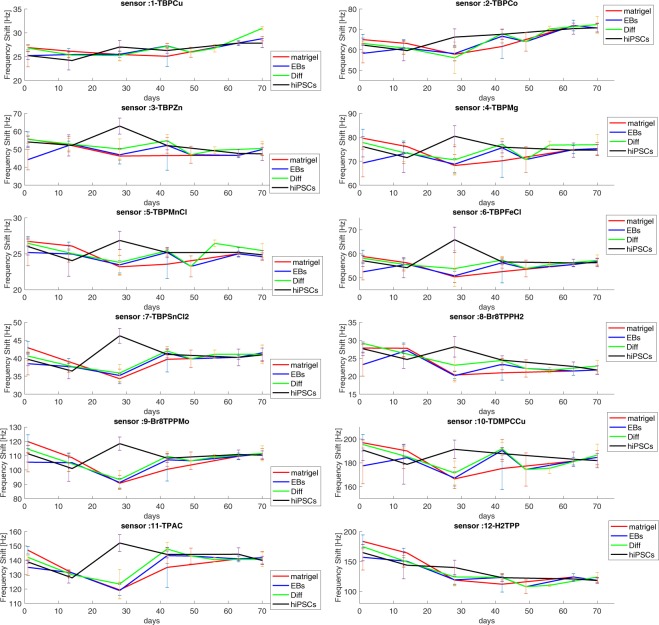
Mean and total dispersion of the sensors signals versus the experiment time.

Figure 4 shows the mean and the total dispersion of the signals of sensors exposed to the cage headspace in the different measurement sessions and for the four groups of mice. Individual sensors signals are shown in figure S2 in the supplementary information file.

Each measure is the average of three consecutive measurements.

Sensor signals are in a good agreement with the GC/MS data. All sensors, except those labelled as 1, 10, and 12, show an increase of their signals at day 28 and only for the mice injected with hiPSCs.

The collective response of the array can be appreciated analyzing the data with the PCA. For a better display of the data, a PCA has been calculated for each measurement session. Far sake of comparison among the different groups, a unique PCA has been calculated with the data collected from day 49 to day 63.

**Figure 5:**
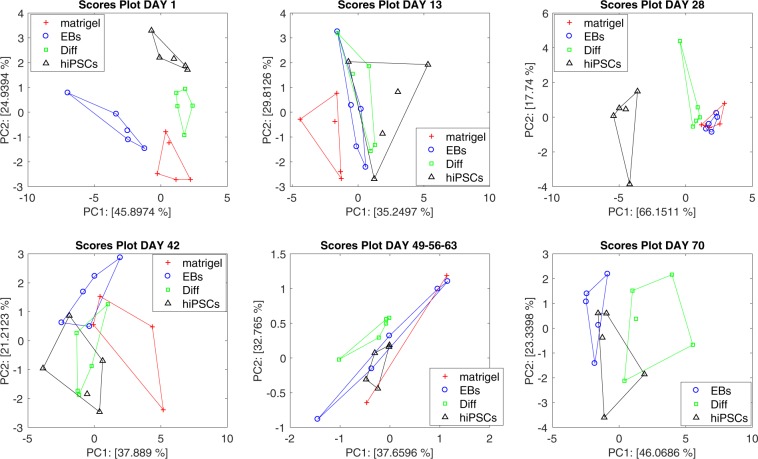
Principal component analysis scores plot of sensors array response to the total volatile metabolites. Separately analysis for each experimental session have been performed. To guarantee a comparison between groups data collected at days 49, 56, and 63 have been merged together.

The plot of the first two principal components is shown in Fig. 5. The total variances explained in these plots are almost constant in the range 58–69%, except at day 28 where the variance explained by the first two principal components reaches the 83%. The variance explained by the first two principal components is a measure of the correlation among the sensors. Thus, the large variance explained at day 28 is explained considering that in this measurement session most of the sensors undergo a common increase of signal for the samples of hiPSCs-injected mice.

In the PCA scores plot at day 1, the four groups of mice are distinct, at day 13 all injected mice are indistinguishable but still different from controls. Any separation among the groups is lost in the rest of the experiment except at day 28 where the group of hiPSCs is clearly segregated from the rest of the data.

The results from floor paper are in agreement with those of the cage air. In principle, the floor paper might also absorb the cage volatile compounds; however, most of the VOCs in the bedding material are supposed to be released by feces and urines.

**Figure 6:**
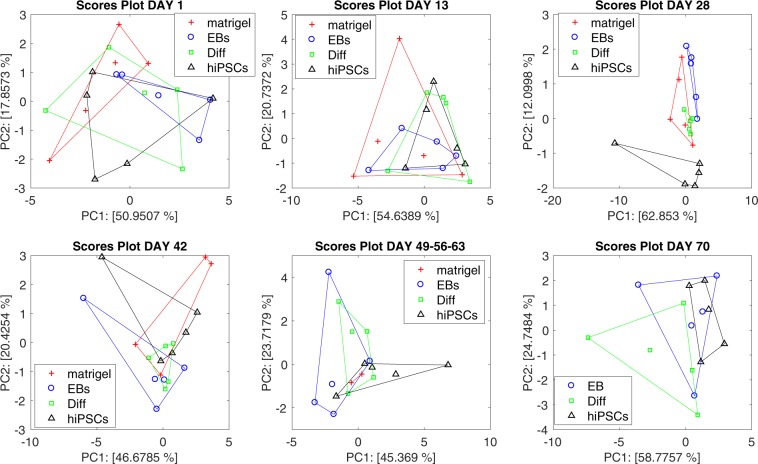
Principal component analysis scores plot of sensors array response to the volatile compounds released by the bedding materials. Separately analysis for each experimental session have been performed. Data collected at days 49, 56, and 63 have been merged together for a better comparison among the groups.

Figure 6 shows the plot of the first two principal components of the PCA calculated with the sensor response to the volatile compounds released by the bedding materials collected immediately after the total cage air measurement. Also in this case, no relevant separations among the four groups of mice is observed except hiPSCs-injected mice at 28 days from the cell injection. However, it is important to note that at day 28, respect to the case of total cage air, the separation of hiPSCs injected-mice occurs along the second principal component. This suggests that the event at day 28 is captured by the bedding materials with less intensity and consensus among the sensors, respect to the total air of the cage.

Differently to VOCs abundance, neither the signals of the individual sensors nor the principal components scores are correlated with the size of the developed teratomas.

## Discussion

As human pluripotent stem cells and their derivatives approach the clinical use, it is of paramount importance to accurately and comprehensively characterize their pluripotency and behavior *in vivo*. The teratoma assay is part of the standard set of quality control and basic characterization used in hiPSCs laboratories. It is performed as part of routine culture evaluation, when new embryonic stem cell (ESC) or induced pluripotent stem cell (iPSC) lines are generated, and when iPSCs are expanded and banked to make working stocks^8,9^. Teratomas are tumors characterized by a rapid growth *in vivo*. Their haphazard mixture of tissues is remnant of all three germ layers, and thus often have semi-semblances of organs, teeth, hair, muscle, cartilage and even bone. These are key characteristics of robust pluripotency and explain why teratoma formation is considered as a hallmark for assessing pluripotency^7^. Upon engraftment, teratoma formation is affected by three main factors: pluripotent capacities of stem cells, cell number, and delivery route^9^.

Teratoma incidence in fact depends on expression levels of stemness genes. Indeed, hiPSCs develop teratoma more efficiently and faster than human embryonic stem cells (hESCs) for both genetic and epigenetic causes^1^.

A clear difference in teratoma-forming propensity has been observed among different hiPSCs^10^. Such variability may be attributed to the different methods of reprogramming, to the differentiation protocols and also to the original somatic target cells used by different groups^11^.

As to the site of cell implantation, some researchers have observed that injection site does affect teratoma formation efficiency^12,13^, while others have found little or no effect^10^. Lastly, regarding the injected cell number, the “critical threshold” that needs to be achieved for teratoma formation has been investigated and estimated about 0.5 × 10^3^–1 × 10^3^ hESCs when injected in subcutaneous dorsal regions^14^.

Several engraftment sites have proven useful for teratoma production; however, the graft site, the number of cells implanted, and the cell preparation has been shown to influence the type of somatic cells found in the teratoma, whether the teratoma is cystic or solid tumor, and its growth rate^10^.

The pluripotency test allows to study hiPSCs behavior within an anatomical microenvironment *in vivo* rather than relying on that of a petri dish. Therefore, teratoma formation assays have demonstrated great potential to significantly help investigators for understanding both basic stem cell biology and tumorigenic potential of therapies prior to their clinical use.

In a previous paper we shown that the volatile fraction of metabolites released by cultured cells is significantly different among the different steps of the differentiation process^4^. In particular, a sharp difference between the hiPSCs and their differentiated stages was found. As a next step, an *in vivo* experiment has been planned to study the volatile metabolites released during the development of implanted cells. The experimental plan has been based on weekly measurements of a cohort of mice injected with three different kind of cells along the successive stages of development: human induced pluripotent cells (hiPSCs), embryoid bodies at 5 days (EBs), and spontaneously differentiated cells (Diff), and an additional control group injected with placebo (matrigel).

Results show that the volatilome of mice is clearly influenced only by hiPSCs injection, highlighting a dramatic change at 28 days after the implant of the abundance of some VOCs and of the magnitude of the sensor signals. In fact, at day 28 a dramatic switch in mice injected by hiPSCs leads to the appearance of a visible and growing grade 3 teratoma.

There is then an unquestionable correspondence between the burst of VOCs abundance and the evolution of hiPSCs into teratoma, with a clear correlation between the size and gravity of teratomas and the abundance of VOCs.

Thus, at day 28 this group of mice becomes clearly distinguishable from the others, and inside them the amount of emitted compound is correlated with the mass of the developed immature teratoma that will evolve into differentiated structure (teeth, hair, muscle, cartilage and even bone).

GC/MS analysis indicates that 13 VOCs are altered at day 28. They are alkanes, methylated alkanes, and aldehydes. Some of these VOCs were previously observed either *in vivo*, in the breath of patients affected by cancer, and in *in vitro* released by various pathogens (Table 2). All these compounds are not specifically connected neither to a disease nor a particular living function, likely they are common volatile metabolites whose abundance increases in correspondence of altered metabolic processes.

**Table 2:** List of GC-MS identified compounds found in more than 70% of the totality of the samples.

#	Name	p-value hiPSCSs vs. others at day 28	Previously Found in:
1	Heptane. 2.4-dimethyl-	0.06	Pseudomonas Aeruginosa^21^ H. Pylori^22^ Lung cancer cells^23^
2	Octane, 4-methyl	0.04	Breath of colorectal cancer^24^ Breath of lung cancer^25^
3*	Decane	0.005	Breath of liver cancer^26^ Pseudomonas Aeruginosa^27^
4*	Octane, 4,4-dimethyl-	0.0003	
5*	Heptane. 2.5.5-trimethyl- ′	0.002	Skin of melanoma^28^
6*	Octane, 2,4,6-trimethyl-	0.0002	
7*	Nonane, 2,6-dimethyl-	0.0003	
8*	Undecane	0.00004	Breath of lung cancer^29^ Breath of neck and head cancer^30^
9*	Octane, 5-ethyl-2-methyl-	0.00007	
10*	Nonanal	0.0008	Plasmodium Falciparum^31^ Breath of lung cancer^32^ Breath of ovarian cancer^33^ Human skin^34^
11*	Nonane, 4,5-dimethyl-	0.0003	Oral cancer^35^
12*	Undecane, 2,9-dimethyl-	<10^−5^	
13*	Decanal	<10^−5^	Breath of ovarian cancer^33^ Human skin^34^
14	Undecane, 3,6-dimethyl-	0.06	
15	3-Ethyl-3-methylheptane ′	0.7	
16	Decane, 2,3,8-trimethyl-	0.05	Colon cancer cells culture^36^
17*	Dodecane. 4.6-dimethyl-′	0.00005	Plasmodium Falciparum^31^
18	Decane, 2,3,5,8-tetramethyl-	0.1	
19*	2,4-Dimethyldodecane	0.0006	
20	Tetradecane	0.3	Plasmodium Falciparum^31^ Human skin^32^
21	Decane, 3-ethyl-3-methyl	0.2	
22	Phenol.3.5-bis(1.1-dimethylethyl)- ′	0.6	

For each compound the p-values between the groups of hiPSCSs injected mice and the others calculated at the 28^th^ day are shown. The compounds with p < 0.01 are marked with a star. In the last column the cases where the VOCs were previously found are indicated.

It is important to remark that the analysis here reported is not comprehensive of the total volatilome. Indeed, the detection of VOCs is influenced by the affinity with the materials of the SPME fibre and the GC column. The methods used in this paper have been imported by the previous *in vitro* study^6^; clearly, a more exhaustive analysis would require the use of multiple SPME fibers and GC columns.

It is interesting to note that the VOCs detected in this experiment are different from those released *in vitro*^4^. Since the experimental set ups were similar, this finding shows that the production of volatile metabolites is strongly influenced by the environment in which cells proliferate. Thus, it is reasonable that *in vitro* and *in vivo*, being completely different environments, elicit different spectra of metabolites.

In this paper, the analysis has been limited to the most frequent VOCs, namely to those compounds that appeared in at least 70% of the samples. In this way, we excluded from the analysis those VOCs that could episodically appear in some implants and then can express some physiological aspects related to the interaction between the implanted cells and the phenotypical features of the host. Furthermore, to limit the analysis of the VOCs common to all the groups and to all the animals may rule out any deviation respect to the common behavior. The investigation of the interaction between stem cells and individual mice was beyond the scopes of this paper, however, future studies could consider how the phenotype of the host influences the evolution of the implanted cells.

The changes of volatile compounds as observed by GC/MS are also captured by the array of sensors. It is important to consider that the responses of sensors could also be influenced by other compounds that have not been detected by the GC/MS. As discussed above, the selectivity of SPME and GC column may hinder other relevant compounds. On the other hand, both alkanes (linear and branched) and aldehydes are compatible with the sensitivity properties of porphyrinoids coated quartz microbalances^15^.

Sensors capture the event of differentiation both in the total air of the cage and in the bedding materials. The measure of these two samples was expected to elucidate which is the more relevant source of volatile compounds, being breath and dejection the major expected sources of VOCs. Results do not provide evidences to answer to this question mainly because the experimental setup did not allow for a net separation of the VOCs source. Indeed, the cellulose paper used as cage floor might also absorb the volatile compounds from the air of the cage, and the dejections clearly contribute to the total air of the cage. However, it is interesting to note that the two datasets differ in which principal component capture the difference between hiPSCs-injected mice and the other groups of mice at day 28. The separation occurs along the first principal component in the case of the total cage air while in the bedding material the separation is along the second principal component. This slight difference suggests that, at day 28, the difference between hiPSCs-injected mice and the other groups of mice affects the bedding materials data, but it is less important than other sources of variability such as the intrinsic differences between animals. This may be due to a random production of dejection that results in a fluctuation of sensors responses.

Eventually, even if this experiment leaves open the question about the source of VOCs, it points out that the measure of the total volatile metabolites can identify the moment of differentiation of hiPSCs. Furthermore, our data show that the hiPSCs implanted in the five mice follow a synchronous development with the time resolution of 1 week defined by the adopted experimental plan.

To our best knowledge, this is the first time that the volatile compounds released during the process of differentiation of pluripotent cells into a tumorigenic form has been observed. The process is signaled by an increase of the abundance of some compounds at which corresponds an increase of the signal of a sub set of sensors of the array.

Although more experiments are necessary to provide a more solid statistical basis and to avoid interference due to the experimental setup (e.g. the origin of the cells and the features of the host), this study indicates that the measure of volatile compounds is a good candidate for a timely and accurate identification of implanted undifferentiated hiPSCs. Furthermore, hiPSCs may be manifested as soon as the differentiation occurs and then well before the appearance of an evident tumor mass.

Finally, since the results of the gas sensor array are in good agreement with those of the GC/MS, this study provides a base for the development of a simple and low-cost instrument for the identification of hiPSCs before and after the *in vivo* implant. The early detection of hiPSCs before the development of tumor may help in designing novel protocols to mitigate risks in regenerative medicine.

Noteworthy the application of this instrument could be very useful in clinical practice for monitoring tumor relapse in those patients underwent surgical procedure. In fact, it has to be noted that the sensors have demonstrated the capacity to detect the presence of pluripotent stem cells (teratogenic cells) soon before the beginning of their spontaneous differentiation. Once this process has started no difference nor quantitative nor qualitative can be detected, because these cells lose their native characteristics.

## Materials and Methods

### Cell culture

Two healthy subjects have been recruited for dermal biopsy. Before participation, informed written consent has been obtained. People enrolled into this study could retract their consent to participate at any time. The project was approved by The Committees on Health Research Ethics of Tor Vergata Hospital (prot. 2932/2017).

Briefly, skin biopsies (6 mm in diameter) have been digested with DISPASE (2 mg/mL), followed by incubation with COLLAGENASE type I (1 mg/mL; SIGMA) and then placed onto 0.1% gelatin (from porcine skin Type A, SIGMA) -coated 35-mm culture plates. Fibroblasts growing out from the dermal tissue have been expanded until passage 2 in primary culture medium DMEM (HyClone) with 15% fetal bovine serum (HyClone), 1mM L-Glutamine (GE Healthcare), 1% penicillin/streptomycin (Carlo Erba), 1% non-essential amino acid solution (GE Healthcare), 0,1 mM β-mercaptoethanol (Gibco).

Successively, human dermal fibroblasts have been reprogrammed using a single lentiviral “stem cell cassette” flanked by loxP sites (hSTEMCCA-loxP), encoding four reprogramming factors (OCT4, SOX2, KLF4, and c-MYC) in a single polycistonic vector, as previously described^16,17^.

hiPSC lines have been expanded, using mechanical passage and placed on irradiated mouse embryonic fibroblast (MEF) feeder layers until lines were well established for at least five passages.

hiPSC lines have been then transferred to Matrigel (BD Biosciences) plates, grown in feeder-free conditions in mTeSR medium (Stem cell Technologies) and propagated to achieve a sufficient number of cells for injection.

For spontaneous differentiation, hiPSCs have been dissociated by collagenase IV/Accutase treatment and plated in hiPSC medium without basic fibroblast growth factor (bFGF) on ultra-low attachment plates. After 7 days in suspension culture, embryoid bodies (EBs) have been transferred to gelatin-coated plates and cultured in DMEM-F12 supplemented with 20% FBS, 1% nonessential amino acid solution, 1mM l-glutamine, and 1% penicillin/streptomycin for another 20 days for spontaneous differentiation into the three germ layers.

### Preparation of cells for transplantation

Five hiPSC lines (3 derived from subject 1 e 2 derived from subject 2) have been treated with collagenase IV/Accutase solution to detach the clones from the plate bottom, pipetting the solution up and down to disaggregate the colonies to single cells. hiPSCs (10 × 10^6^ cells per injection) have been resuspended in 50 μl of PBS and an equal volume of chilled Matrigel, then placed on ice until injection.

EBs, derived from the same hiPSC lines above described and cultured in suspension for 5 days, have been dissociated using Trypsin-like enzyme (TrypLE, Invitrogen). Single cell suspension (5 × 106 per injection) has been obtained in 50 μl of PBS and an equal volume of chilled Matrigel, then placed on ice until injection.

EBs have been spontaneously differentiated obtaining cells of all three germ layers after 20 day of culture in adhesion. Adherent cells have been harvested by trypsin-EDTA treatment and resuspended at a concentration of 5 × 10^6^ in 50 μl of PBS (per injection). Cell/PBS mixture have been kept on ice until injection with 50 μl of Matrigel.

### Cell transplantation into Nude mice

All procedures involving mice and care have been conducted in accordance with the ethical standards, according to the Declaration of Helsinki, in compliance with our institutional animal care guidelines and following national and international directives (Italian Legislative Decree 26/2014, Directive 2010/62/EU of the European Parliament and of the Council) (protocol n. 954/2016-PR).

CD-1 Nude mice (Charles River Laboratories, Wilmington, MA) with 5-week-age (n = 5 per group) were housed in the Interdepartmental Service Centre - Station for Animal Technology, University of Rome “Tor Vergata” (Italy) at a constant temperature of 20 ± 2 °C, relative humidity of 50 ± 10%, on a 12:12 h light/dark cycle and ventilation 10–20 times/h. Mice were housed in filter-top cages; irradiated laboratory rodent pellet diet (4RFN; Mucedola srl, Italy) and municipal tap water were provided to the animals *ad libitum*.

The animals have been anesthetized, before cell engraftment, according to respective institution’s animal use protocol. General anesthesia has been achieved using an intraperitoneal injection of tiletamine/zolazepam (40 mg/kg) (Zoletil 100, Virbac, Italy) associated to xylazine (15 mg/kg) (Rompun, Bayer, Italy) and the animals were maintained under anesthesia, preferably on a 37 °C heat pad, for 10–20 minutes after injection and monitored during the duration of anesthesia to avoid death from overdosing and hypothermia from long periods of knockdown.

In order to enhance post-injection cell engraftment, the animals have been awaked only after the Matrigel/PBS/cell mixture has solidified in the localized transplantation site.

hiPSC lines, EBs day 5 and spontaneously differentiated cells have been transplanted subcutaneously (10 × 10^6^ of hiPSCs, 5 × 10^6^ of floating EBs or differentiated cells/mouse) in the right flank of mice (n = 5 per group). A group of mice have been injected only with Matrigel as a control.

Body weights and tumour sizes have been measured once a week immediately before the volatile compounds sampling. Tumour size was measured in two dimensions by a caliper. Volumes (V) have been calculated with the following formula:

$${\rm{V}}=({\rm{L}}\ast {{\rm{w}}}^{2})/2{\rm{w}}$$

where V is the volume, L the length and w the width. Teratomas became measurable 4 weeks post-injection and were removed after 10 weeks, when reached approximately 1–2 cm^3^ in total volume. Mice have been sacrificed after 10 weeks and their major organs harvested.

The experimental procedure is synthetically reported in Table 1.

### Histological evaluation

Tumor inoculation sites and organs were taken, fixed with 10% neutral-buffered formalin and then embedded in paraffin. Subsequently, the sections (4µm-thick) were stained with hematoxylin and eosin (H&E). Microscopic evaluation was performed to characterize and classify teratomas^18,19^ and the presence of possible metastases in the organs. Representative images were acquired by using a digital camera (E600 Eclipse, Nikon).

### Volatile compounds sampling

In order to sample the VOCs, animals were kept for 30 minutes without food and then moved in the experiment room and placed in a polypropylene box (KIS T box XXS- ABM Italia S.p.A). The box was large enough to allow for a normal physical activity. The lid of the box was endowed with an inlet suitable for the insertion of either the Solid Phase Micro-Extraction (SPME) sampler or the gas sensor array sampling tube. The SPME fibre was a 50/30 μm Divinylbenzene/Carboxen/PDMS (SUPELCO, Bellefonte, PA, USA). The fibre was kept in the sampling box for 1 h.

To standardize the headspace formation, The VOCs collection began 15 minutes after the mouse entered the cage. The temperature and humidity of the experiment room were kept constant during the whole experiment.

The box floor was coated with filter paper sheets (Biosigma srl) to collect solid and liquid dejection. The layer was removed after each measurement, placed in a sealed vial and kept refrigerated at T = −20 °C until the analysis.

### Gas Chromatography Mass Spectroscopy

The experimental setup of GC/MS was the same used to measure the volatile compounds released by stem cells *in vitro*^6^.

SPME fibres have been analyzed over three hours after their collection with a GC/MS (Shimadzu GCMS-QP2010, Kyoto, Japan).

The GC column was an EQUITY-5 capillary column (poly(5% diphenyl/95% dimethyl siloxane) phase (SUPELCO, Bellefonte, PA, USA). The size of the column was 30 m length × 0.25 mm I.D. × 0.25 μm thickness.

The VOCs adsorbed in the SPME were desorbed from the fiber in splitless injection mode at 250 °C for 3 minutes in the GC injection port. VOCs were separated on the GC column using an initial oven temperature of 40 °C for 5 minutes, then increased by 7 °C/min to 220 °C, afterwards ramped by 15 °C/min to 300 °C that was held for 3 min (total runtime: 39 min). Ultra-high purity helium has been used as carrier gas, working in linear velocity constant mode, with a carrier gas pressure of 24.9 kPa, total flow of 5.9 mL/min, column flow of 0.7 mL/min and linear velocity of 30.2 cm/s.

The mass spectrometer was a single quadrupole analyzer in electron ionization mode and was set to record between 40 and 450 amu in the full scan mode. The temperature of transfer line and ion source was 250 °C. The detector voltage was set at 0.7 kV. GC-MS data were analyzed using the section GC-MS post-run analysis of the GCMS solutions software (version 2.4, Shimadzu Corporation).

Compounds have been putatively identified using both NIST 127 and NIST 147 libraries. The identity of the compounds involved with the hiPSC differentiation has been confirmed comparing the mass spectra with those of pure standard obtained from Sigma Aldrich. Pure compounds were used as received without any further purification.

### Gas sensor array

The gas sensor array was an ensemble of twelve quartz microbalances (QMB). In these sensors, a mass change (Δm) on the quartz surface results in frequency changes (Δf) of the electrical output signal of an oscillator circuit at which each sensor is connected. In the low-perturbation regime, Δm and Δf are linearly proportional^20^. QMBs had a fundamental frequency of 20 MHz, corresponding to a mass resolution of the order of a few nanograms.

The sensing materials were the same solid-state layers of porphyrins and corroles used to measure the VOCs from stem cells *in vitro*^4^.

The sensor system used in these experiments was the last version of a series of instruments designed since 1996 at the University of Rome Tor Vergata. The gas sensors are complemented by temperature and relative humidity sensors. Each QMB is connected to an oscillator circuit, the frequencies of the oscillators outputs are measured taking advantage of a temperature compensated reference quartz that allows for a frequency resolution of 0.1 Hz. Electronics is implemented in a FPGA. Gaseous samples delivery is controlled by a miniature diaphragm pump (0–200 sccm). The instrument is connected and powered via a single USB connection. Functions and the data acquisition are controlled with an in-house software running in Matlab.

The baseline of sensor signals was measured in a constant flow of reference air made filtering ambient air with a CaCl_2_ trap. The difference of the sensors signals taken in reference air respect to sample air was used as the sensor response.

The cage air was sampled for three minutes at the constant flow of 75 sccm.

The cage floor covering material was collected immediately after the cage air measurement. The paper sheets were transferred in vials and stored at −20 °C. Vials caps were endowed with air inlet and outlet connections. Before measurement, the vials were kept at 30 °C for 15 minutes and then the headspace was transferred in the sensors chamber by a stream of reference air generated as described above.

### Data analysis

The statistical differences between the abundances of the VOCs from mice inoculated with hiPSCs and the other groups were evaluated with the a Kruskal-Wallis rank sum test.

The absolute abundances of GC-MS identified peaks and the sensor responses were arranged in matrices and then analysed with multivariate data analysis. Principal component analysis (PCA) was calculated on autoscaled data matrices^7^. In autoscaling each variable of the matrices (either GC/MS peaks or sensors signals) were normalized to null mean and unitary variance. All calculations were performed in Matlab R2017a, PCA was calculated with the Statistics and Machine Learning toolbox of Matlab.

## Electronic supplementary material

Supplementary Information
