# Supplementary material for: Volatile compounds emission from teratogenic human pluripotent stem cells observed during their differentiation in vivo
Source: Sci Rep. 2018 Jul 23;8:11056. doi: 10.1038/s41598-018-29212-0 (PMC6056464; doi:10.1038/s41598-018-29212-0)
Supplement: Supplementary file 1 — Supplementary Information [file 41598_2018_29212_MOESM1_ESM.pdf]

Volatile compounds emission from teratogenic human pluripotent stem cells observed during their differentiation *in vivo*

Rosamaria Capuano[1], Paola Spitalieri[2], Rosa Valentina Talarico[2], Alexandro Catini[1], Ana Carolina Domakoski[3], Eugenio Martinelli[1], Maria Giovanna Scioli[2], Augusto Orlandi[2], Rosella Cicconi[4], Roberto Paolesse[3], Giuseppe Novelli[2], Corrado Di Natale[1§] and Federica Sangiuolo[2]

[1] Department of Electronic Engineering, University of Rome Tor Vergata, Via del Politecnico 1, 00133 Rome, Italy

[2] Department of Biomedicine and Prevention, University of Rome Tor Vergata, Via Montpellier 1, 00133 Rome, Italy

[3] Department of Chemical Science and Technology, University of Rome Tor Vergata, Via della Ricerca Scientifica, 00133 Rome, Italy

[4] Centro Servizi Interdipartimentale STA, University of Rome Tor Vergata, Via Montpellier 1, 00133 Rome, Italy

§ correspondence to: dinatale@eln.uniroma2.it and sangiuolo@med.uniroma2.it

SUPPLEMENTARY INFORMATION FILE

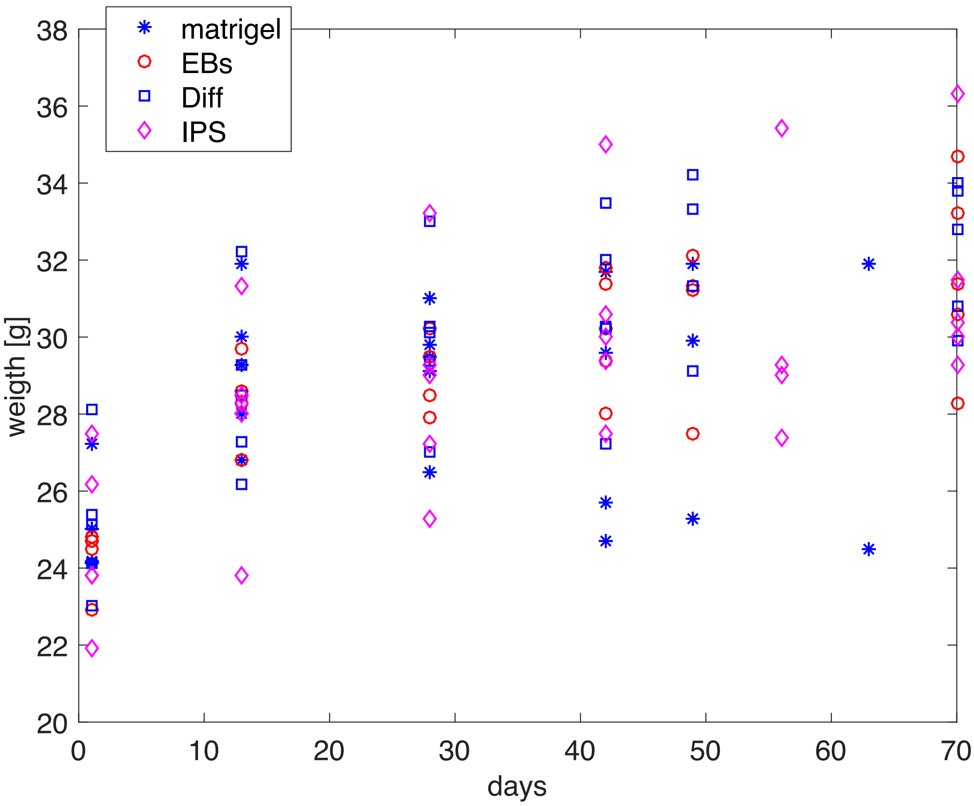

Figure S1: Evolution of the weight of mice along the experiment. Mice are labelled with their membership to the four groups as indicated in the legend.

Table S1: List of the VOCs identified in the mice cage headspace. The percentage of occurrence indicate the percentage of samples where the compound has been found. In the table the putative identification based on the database matching with the libraries NIST 127 and NIST 147 is shown, the percentage of similarity with the database is reported for each compound. Those compounds that occurred in more than 70% of the samples were further identified by standard comparison and used in the following analysis.

Retention time (minutes)	Similarity	Identification	Occurrence
4.237	80%	Ethanol, 2-(1-methylethoxy)-	1.8%
5.699	99%	Toluene	1.8%
7.016	75%	4-Hepten-3-one. 5-methyl-	5.4%
7.407	92%	Heptane. 2.4-dimethyl-	84.6%
8.717	93%	Octane, 4-methyl	100.0%
9.056	88%	Pyruvic acid, butyl ester	0.9%
9.455	82%	styrene	17.1%
9.811	90%	Nonane	48.6%
10.317	91%	Acetyl valeryl	2.7%
10.534	91%	Propanoic acid, anhydride	0.9%
10.662	89%	2,2,5-Trimethylhexan-4-one	35.1%
10.785	91%	.alpha.-Pinene	8.1%
11.650	92%	Decane	100.0 %
11.993	93%	Octane, 2,4,6-trimethyl-	100.0%
12.370	90%	Heptanol	0.9%
12.857	93%	Heptane. 2,5,5-trimethyl-	93.6%
12.987	93%	Octane, 2,4,6-trimethyl-	100.0%
13.200	92%	Nonane, 2,6-dimethyl-	100.0%
13.293	85%	Limonene	5.4%
13.481	73%	Butanoic acid, 1,1-dimethylethyl ester	5.4%
13.773	79%	7-Exo-ethyl-5-methyl-6,8-dioxabicyclo[3.2.1]oct-3-ene	16.2%
13.931	88%	Propanoic acid, 2-methyl, anhydride	16.2%
14.097	92%	Undecane	100.0%
14.230	94%	Octane, 5-ethyl-2-methyl-	96.3%
14.716	86%	1-Octanol, 3,7-dimethyl-	11.7%
15.006	89%	Nonane, 5-butyl-	14.4%
15.168	93%	Nonanal	100.0%
15.300	86%	Nonane, 4,5-dimethyl-	72.0%
15.749	88%	3,4-Hexanedione, 2,2,5-trimethyl-	2.7%
16.570	94%	Undecane, 3,7-dimethyl-	52.2%
16.839	93%	Undecane, 2,9-dimethyl-	100. 0%
17.364	94%	Dodecane	53.1%
17.486	92%	Decanal	98.1%
17.674	96%	Undecane, 3,6-dimethyl-	79.2%
17.843	94%	Dodecane, 4-methyl-	48.6%

18.180	90%	Undecane, 3,8-dimethyl-	4.5%
18.314	90%	Decane, 2,3,5-trimethyl-	59.4%
18.537	91%	3-Ethyl-3-methylheptane	90.0%
18.611	94%	Hexane, 2,4,4-trimethyl-	13.5%
18.725	90%	Decane, 2,3,8-trimethyl-	100.0%
18.976	73%	Dodecane, 2,6,11-trimethyl-	5.4%
19.069	90%	Dodecane. 4.6-dimethyl-	95.4%
19.239	93%	Dodecane, 2,7,10-trimethyl-	68.4%
19.352	91%	Decane, 2,3,6-trimethyl	55.8%
19.518	89%	Decane, 2,3,5,8-tetramethyl-	74.7%
19.621	90%	2,4-Dimethyldodecane	91.8%
19.772	83%	Hexyl octyl ether	7.2%
21.397	86%	Decane, 6-ethyl-2-methyl-	48.6%
21.835	89%	Tetradecane	74.7%
22.376	90%	Decane, 3-ethyl-3-methyl	70.2%
22.547	88%	Dodecane, 2,6,10-trimethyl-	69.3%
22.787	81%	1,7-Dimethyl-4-(1-methylethyl)cyclodecane	11.7%
23.052	87%	Pentadecane	49.5%
23.381	84%	Phenol. 3.5-bis(1.1-dimethylethyl)-	80.1%
26.685	90%	heptadecane	27.9%

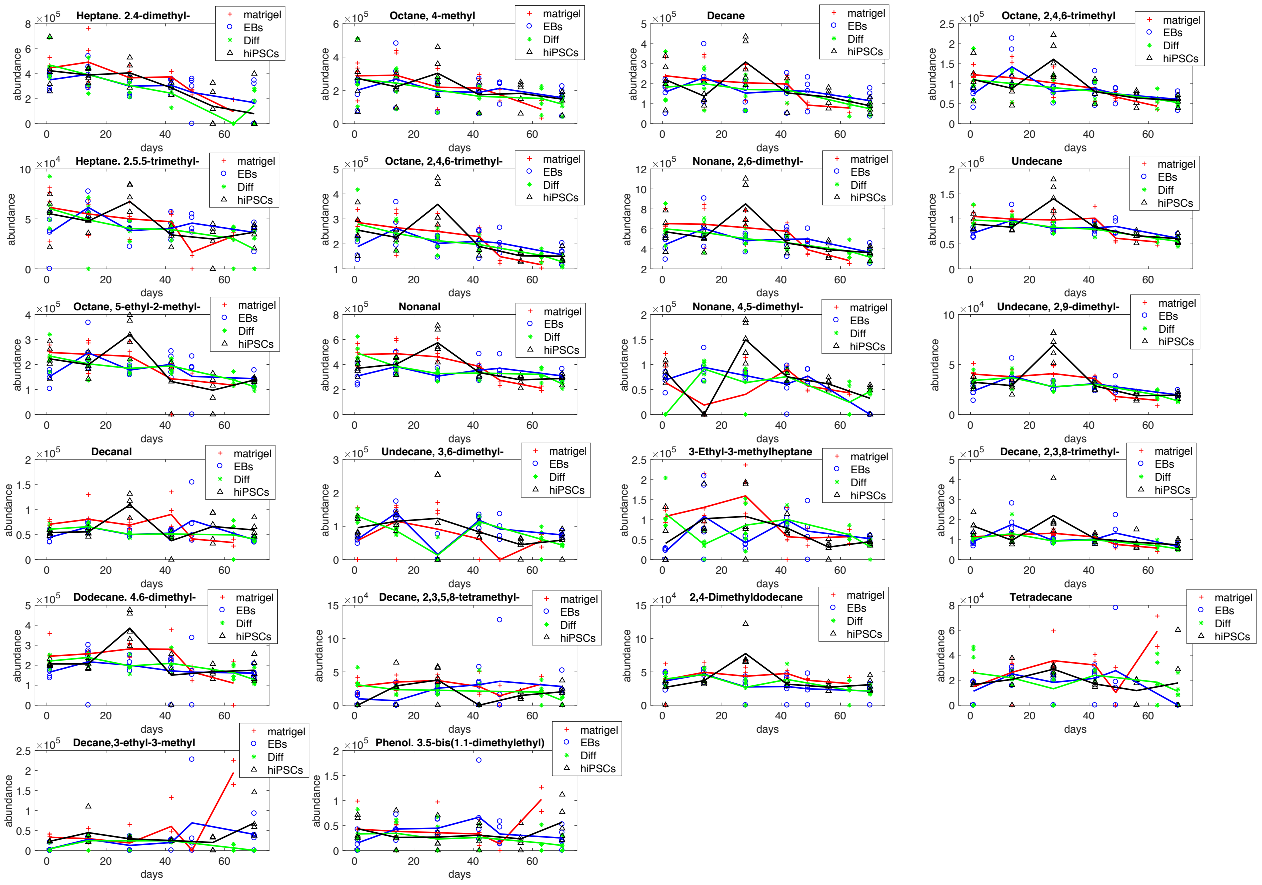

Figure S2: Abundance of the most recurrent VOCs versus the days since the inoculation. Points are the values for each mouse, Thick lines show the average abundance in each group.

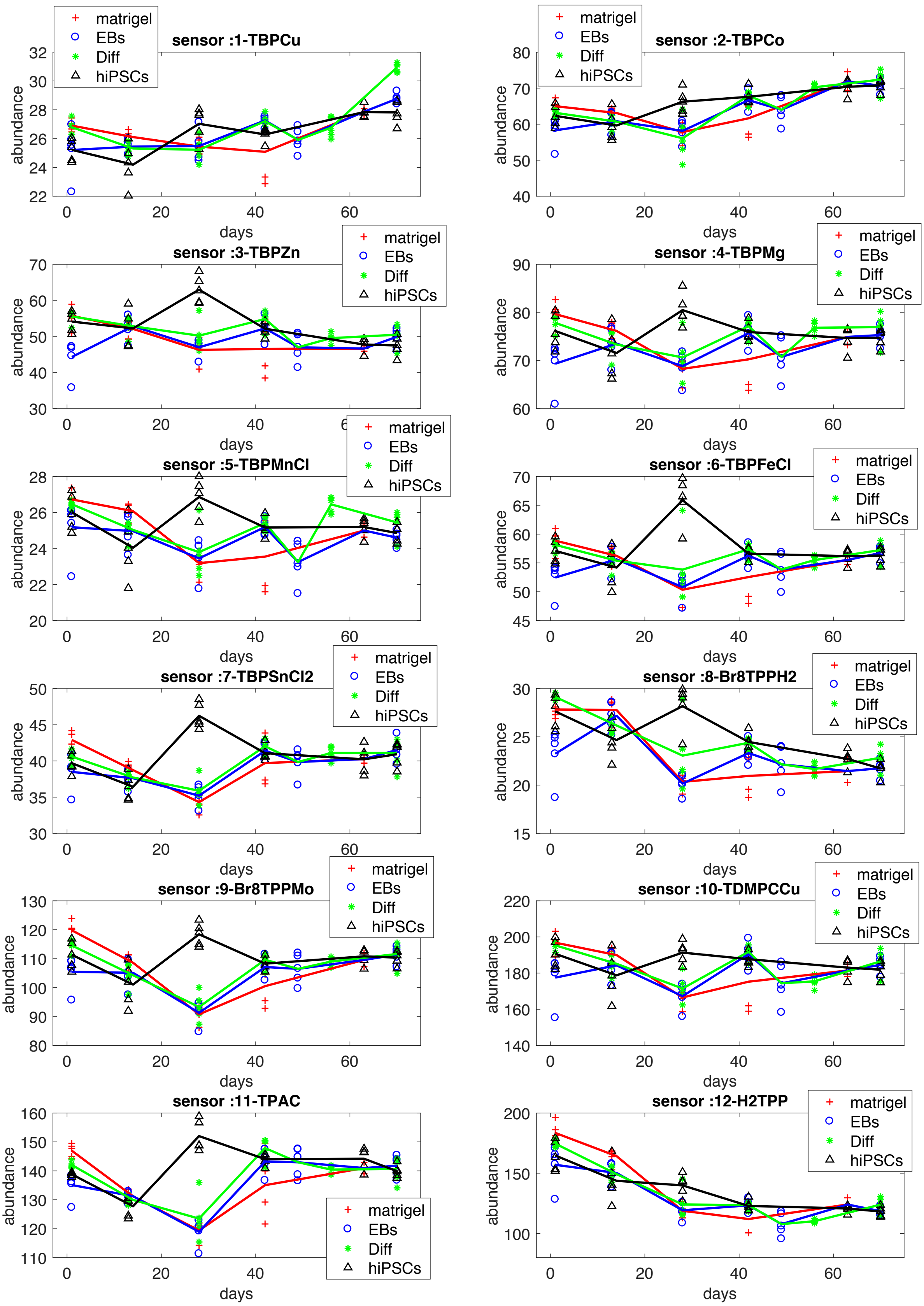

Figure S2: Sensors signals versus the experiment time. Each point corresponds to a mouse. Thick lines show the average signal of each group
